# A residual-based deep learning approach for ghost imaging

**DOI:** 10.1038/s41598-020-69187-5

**Published:** 2020-07-22

**Authors:** Tong Bian, Yuxuan Yi, Jiale Hu, Yin Zhang, Yide Wang, Lu Gao

**Affiliations:** 1 School of Science, China University of Geosciences, Beijing, 100083 China; 2 School of Information Engineering, China University of Geosciences, Beijing, 100083 China; 3 School of Remote Sensing and Information Engineering, Wuhan University, Wuhan, 430072 China

**Keywords:** Optics and photonics, Physics

## Abstract

Ghost imaging using deep learning (GIDL) is a kind of computational quantum imaging method devised to improve the imaging efficiency. However, among most proposals of GIDL so far, the same set of random patterns were used in both the training and test set, leading to a decrease of the generalization ability of networks. Thus, the GIDL technique can only reconstruct the profile of the image of the object, losing most of the details. Here we optimize the simulation algorithm of ghost imaging (GI) by introducing the concept of “batch” into the pre-processing stage. It can significantly reduce the data acquisition time and create reliable simulation data. The generalization ability of GIDL has been appreciably enhanced. Furthermore, we develop a residual-based framework for the GI system, namely the double residual U-Net (DRU-Net). The imaging quality of GI has been tripled in the evaluation of the structural similarity index by our proposed DRU-Net.

## Introduction

Ghost imaging (GI) is an unconventional imaging method compared with traditional optical imaging methods. It was first proposed as a quantum entangled phenomenon by making use of the entangled two-photon light source generated by the spontaneous parameter down-conversion (SPDC) process^1^. However, GI was demonstrated to be accomplished by classical incoherent light source soon later^2^. It led to controversy focus on whether the quantum entangled is necessary for the GI system. The GI system contains two distributed light beams, which are the test beam and the reference beam. In the test beam, the light illuminates the object directly and then be collected into a bucket measurement. In the reference beam, the light travels freely to a high-resolution detector without interacting with the object. The image can then be reconstructed through the correlation measurement between the two light beam signals^3^. In terms of computational ghost imaging (CGI), the reference beam becomes unnecessary as one can obtain the image by calculating the correlation between the test beam intensity and the knowledge of the random patterns displayed on the spatial light modulator (SLM)^4–6^.

CGI usually needs a large number of random patterns to avoid noise disturbance to achieve images of high resolution. This requirement leads to a long data acquisition time, which is the main issue preventing CGI from practical use. Improved correlation method such as normalized ghost imaging^7^ and differential ghost imaging^8^ was later proposed to increase imaging efficiency. Notably, the Gerchberg–Saxton algorithm and compressive sensing ghost imaging (CSGI) regard GI as an optimization problem. In^9^, the Gerchberg-Saxton-like technique takes the integral property of Fourier transform into full consideration to provide a different perspective of image reconstruction of GI. CSGI can reduce measurement times and boost imaging quality^10–14^.

Recently, deep learning (DL) has achieved widespread use in various fields, such as image denoising, image inpainting, natural language processing, to name a few^15^. DL has also made a comprehensive application in computational imaging^16^ such as digital holography^17^, lensless imaging^18^, imaging through scattering media and turbid media^19–21^, optical encryption^22,23^ and ghost imaging^24–28^. The term of ghost imaging using deep learning (GIDL) was first proposed by Lyu et al.^24^. Their research shows that GIDL can accomplish the reconstruction of binary images of high quality, even with significantly less sampling rate $\beta = N / M$, where *N* represents the number of measurements, and *M* stands for the number of pixels of the image. GIDL generally pairs up thousands of GI images as inputs and their corresponding images of the object as targets to train the deep neural network (DNN). Here we use the term “GI image” to represent the image reconstructed by traditional ghost imaging. GIDL can reconstruct objects from severely disrupted images in which the object can hardly be recognized. In the evaluation of the peak signal to noise ratio (PSNR), structural similarity (SSIM) index, and root mean squared error (RMSE), GIDL shows excellent results^25,26,28^. Also, GIDL needs far less processing time than non-deep-learning-based methods^25^. Previous research demonstrates that deep-learning-based solvers for general computational imaging can be trained by simulation data to make it more practical^28^.

GIDL usually takes a large number of images of the object as ground-truth, and then they are multiplied with a series of random patterns to create corresponding GI images as the network inputs. However, among most GIDLs proposed so far, the same set of specific random patterns is used unchangingly in both the training and test set. This drawback may lead to unsatisfactory performance in practical applications with weakened generalization ability^29^. Here we optimize the simulation algorithm of GI with batch processing of different random patterns to create datasets similar to real physical condition. We apply this optimized simulation algorithm in DNNs for the GI scenario, and the generalization ability of existing GIDL is enhanced obviously. In order to improve the imaging quality of GIDL, we make DNN learn the residual image but not the image of the object. We divide the residual of GI into two and further develop a double residual (DR) framework by learning two different kinds of residual images. Through combining the DR framework with the U-Net, we propose a new GIDL, namely the double residual U-Net (DRU-Net). The image quality has been significantly improved with the triple SSIM index.

## Simulation

### Optimized simulation algorithm with batch processing

**Figure 1:**
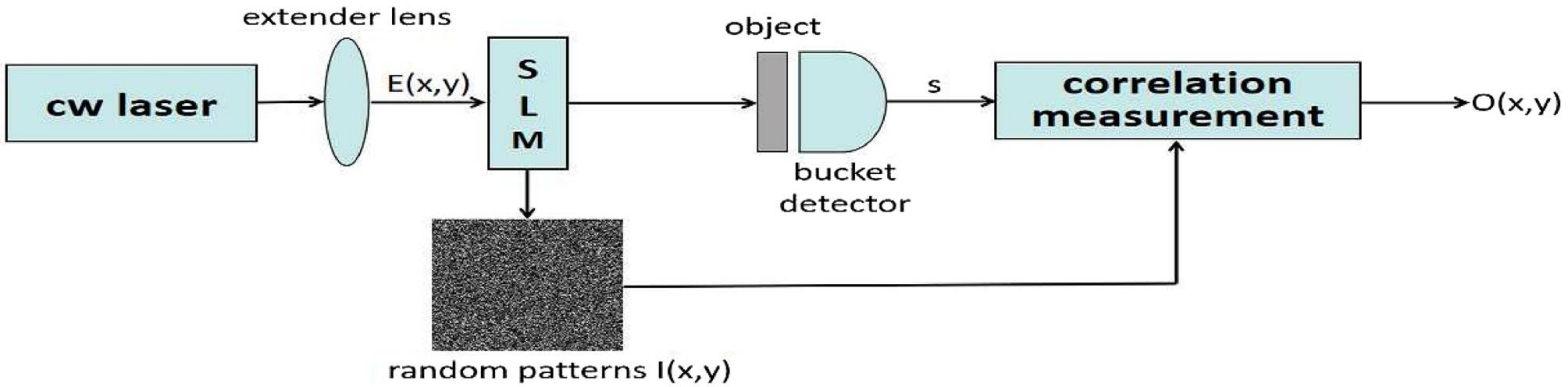
Schematic illustration of the CGI setup.

The configuration for CGI is shown in Fig. 1. Here we simulate it on the computer. A series of random patterns is created and multiplied with the image of the object. It is then summed up as a single pixel to accomplish the bucket measurement. Images can be reconstructed by the correlation between the bucket measurement point intensity in the test beam and the random patterns in the reference beam. The bucket measurement function is given by

1 $$\begin{aligned} T_{GI}(x,y) = \left\langle S_mI_m(x,y) \right\rangle - \left\langle S_m \right\rangle \left\langle I_m(x,y) \right\rangle , \end{aligned}$$

where $\langle \cdot \rangle$ denotes the average of the multiple measurements.

The pseudothermal light random patterns are generated through the simulation to train the network^28^. In the plain serial simulation algorithm, images are processed one by one to achieve the corresponding images of GI. And creating a different set of random patterns for each image will lead to huge input/output (I/O) overhead. Since DL usually needs datasets of thousands of images, it will take tens of hours to generate them with the plain serial simulation algorithm. In previous research, a general method is to make use of the same set of random patterns to generate datasets?. It can reduce both the random pattern generating time and I/O overhead at the cost of the reliability of data and network’s generalization ability. Here we optimize the simulation with batch processing to create more reliable data with different sets of random patterns. We introduce the concept of batch in stochastic gradient descent (SGD)^30^ into the pre-processing stage to make full use of the Compute Unified Device Architecture (CUDA). Images are divided into batches and then combined in the same batch into a 3-dimensional (3D) array. Each 3D array will be taken as the smallest operation unit and a different set of random patterns are created for the specific 3D array. Thus, images in the same batch share the same set of random patterns, whereas different sets of random patterns are used among batches. The GI images are achieved from the dot product of the 3D object distributions and their corresponding random patterns. By setting batch size as 256, 512 or even larger number, our optimized simulation algorithm significantly reduces the I/O overhead. We then normalized the GI simulation data according to the formula as

2 $$\begin{aligned} T_{GI}\left( x,y\right) =\frac{T_{GI}\left( x,y\right) -{T_{GI}}_{\min }\left( x,y\right) }{{T_{GI}}_{\max }\left( x,y\right) -{T_{GI}}_{\min }\left( x,y\right) } \end{aligned}$$

In this way, the generalization ability of GIDL has been improved. Our optimized algorithm can deal with grayscale image as well as binary images. Both the plain serial simulation algorithm and optimized simulation algorithm are implemented by PyTorch, which is a useful python machine learning library. It has been optimized for matrix, hence most of the matrix calculation time can be saved. For the training set of $128 \times 128$ pixels images, it takes almost 28 h to process them with the plain serial simulation algorithm but only 23 minutes with our optimized simulation algorithm by the laptop. The calculation efficiency is increased by 70 times.

### Proposed double residual learning method

In existing GIDLs, images of the object are reconstructed end-to-end from GI images. This process can be expressed as

3 $$\begin{aligned} O(x,y) = F \{ T_{GI}(x,y) \}, \end{aligned}$$

where *O* stands for the output of the network, and $F \{ \cdot \}$ represents the network that maps the GI image to the corresponding image of the object. This specific training process is given by

4 $$\begin{aligned} F = \mathop {\arg \min }_{\theta \in \Theta } \sum _{i = 1}^{K} L(T^i(x,y), F_{\theta } \{ T^i_{GI}(x,y) \} ) + W(\theta ), \end{aligned}$$

where $\Theta$ is the set of network parameters, $L(\cdot )$ is the loss function used to measure the distance between the network outputs and their corresponding images of the object, and the superscript *i* denotes the ith in-out pair. Here we have $i = 1 \dots K$, enumerating the total *K* in-out pairs. The last term, $W(\theta )$, means the regularization of parameters in case of overfitting^29,31^.

In recent years, we have witnessed the widespread applications of residual learning in many image processing fields^32,33^. Inspired by the DnCNN^34,35^, we make DNN learn the residual image but not the image of the object. When applied in GIDL, the residual, denoted as *Res*, can be written as

5 $$\begin{aligned} Res(x,y) = T(x,y) - T_{GI}(x,y), \end{aligned}$$

DnCNN is a deep residual network able to handle several general image denoising tasks, including Gaussian denoising, SISR and JPEG image deblockin^34,35^. It can yield better results than other state-of-the-art methods. Using *R* to denote the DnCNN, the training process can be formulated as

6 $$\begin{aligned} R = \mathop {\arg \min }_{\theta \in \Theta } \sum _{i = 1}^{K} L(Res^i(x,y), R_{\theta } \{ T^i_{GI}(x,y) \}). \end{aligned}$$

In this way, we can express the reconstructed image as

7 $$\begin{aligned} O(x,y) = T_{GI}(x,y) + R \{ T_{GI}(x,y) \}. \end{aligned}$$

**Figure 2:**
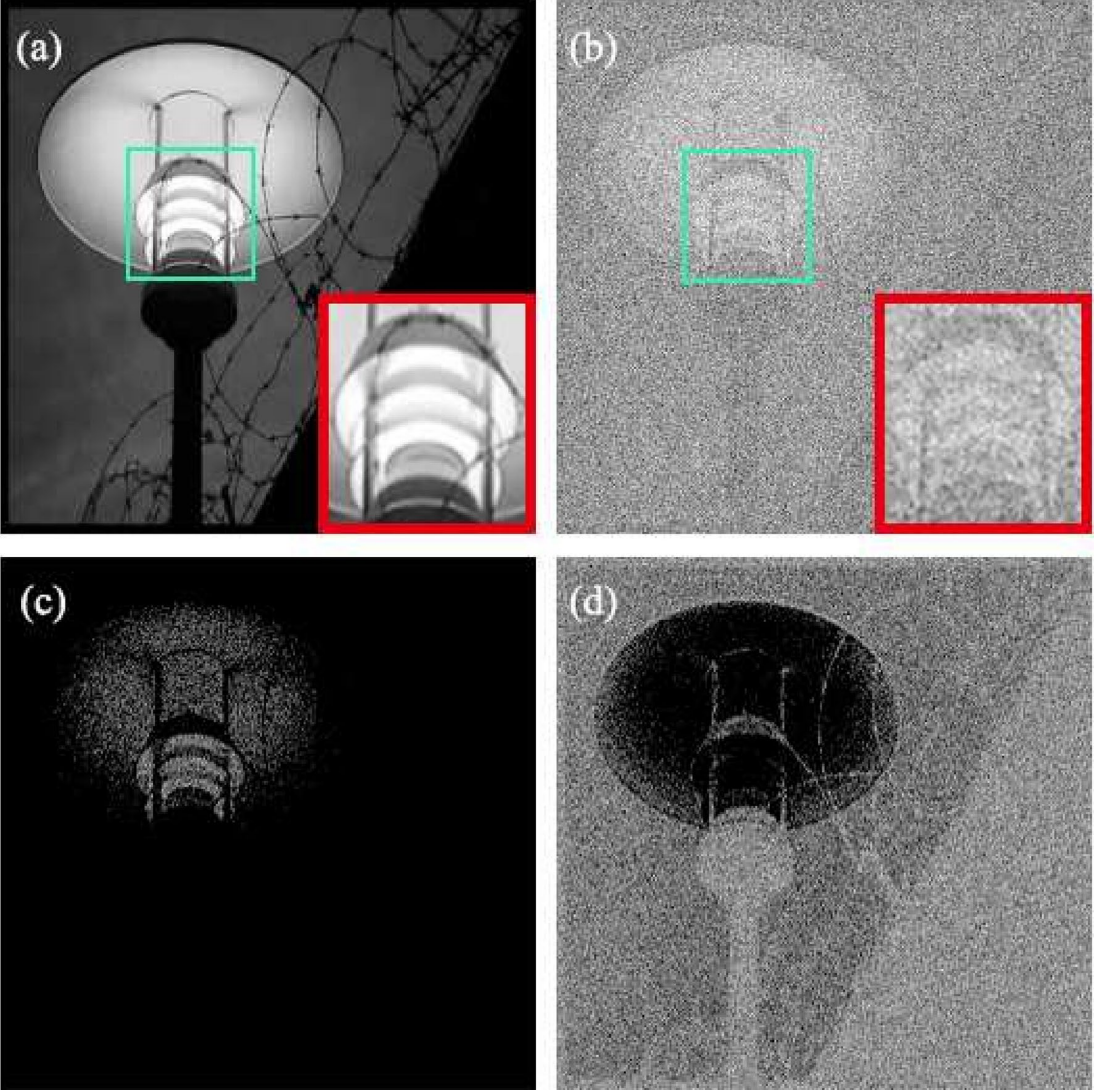
A comparison between image of the object and GI simulation. The image of the object is shown in (**a**), while the corresponding GI image is shown in (**b**), the up-Res image is shown in (**c**) and the down-Res image is shown in (**d**).

The residual mapping is usually easier to optimize than the original mapping as the residual image has small enough pixel values compared to those of the object’s image^33,34^. To the extreme, if the identity mapping is optimal, it would be easier for the network to push the residuals to zero than fit the identity mapping. However, GI is an exception as its residual is of too high value making it hard to learn. In GI images, such as shown in Fig. 2b, the correlation measurement leads to the blurred image with the averaged pixel value. It turns out that the residual between Fig. 2a,b is in the range of − 209 to 145. The interval length exceeds the upper limit of the grayscale. It means that the residual is much more complex than the image of the object. Thus residual learning does not appear adaptive to GI. Here we produce two different kinds of residuals, namely the up-residual (up-Res) and down-residual (down-Res), to reduce the range of the pixel value to make it consistent with the classical nature of residual learning. The learning process becomes easier by dividing the residual into two parts. Figure 2c,d show the up-Res and down-Res images, respectively.

**Figure 3:**
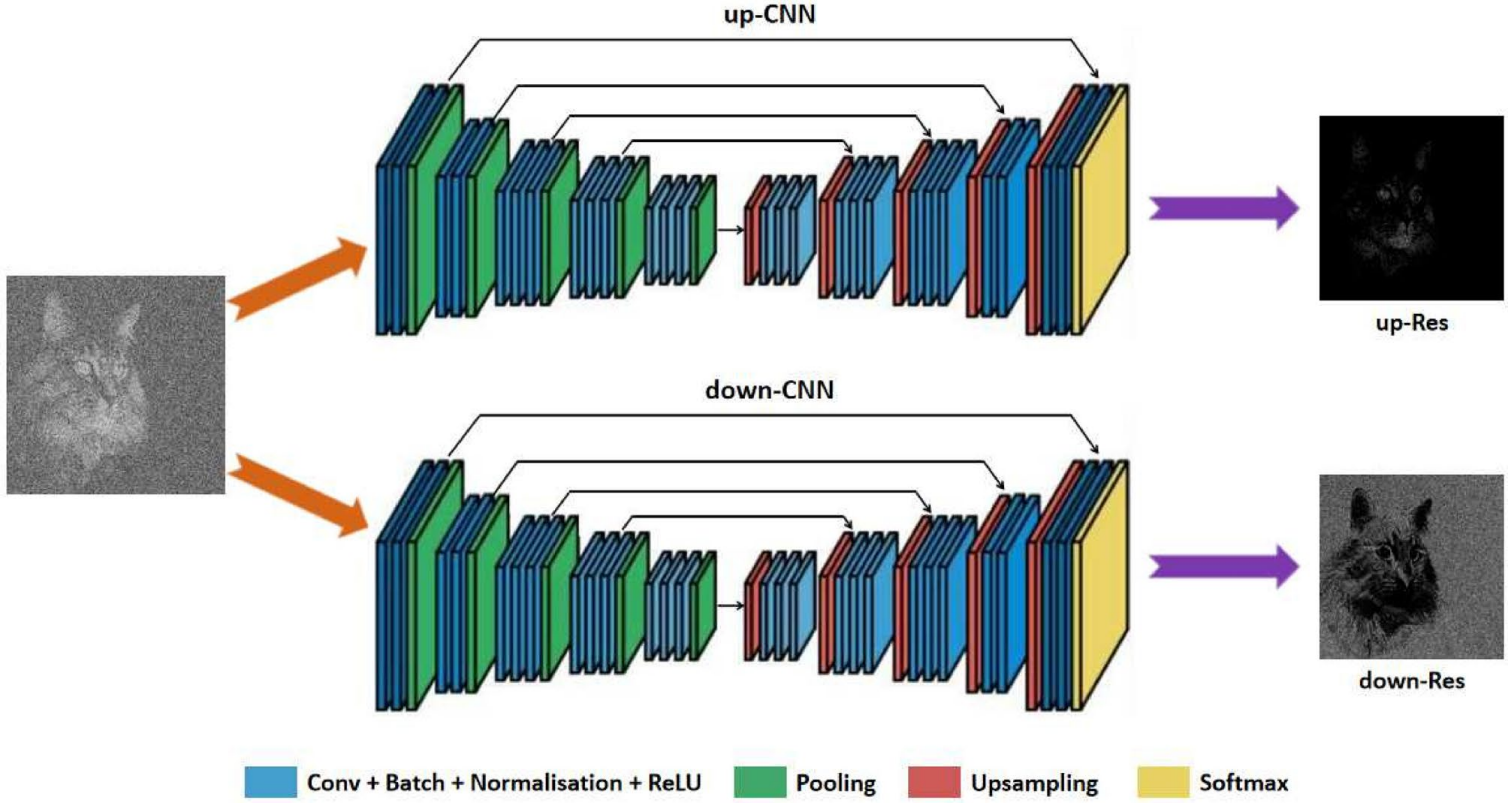
Detailed schematic illustration of the DRU-Net. The image of the object is multiplied with a series of, say *M*, random patterns. After that, we can get a real-valued 2-dimensional matrix whose size is the same as the image of the object. We then normalize the data and feed them to the DRU-Net. The DRU-Net consists of two networks, which are the up-CNN and down-CNN. The up-CNN has to reconstruct the up-Res image, and the down-CNN aims to learn the down-Res image.

The main body of the DR framework consists of two CNNs. We refer to our proposed network as double residual U-Net (DRU-Net). The U-Net is a deep CNN with a U-shaped structure, where the max-pooling layers and the up-sampling layers are symmetrical to each other^36^. It was initially designed for image segmentation, but it can also be used in image denoising problem and ghost imaging^26^. Its variant ResUNet-a has also achieved significant improvement in remotely sensed data^37^. As schematically outlined in Fig. 3, one CNN is trained with up-Res images, and the other is trained with down-Res images. The DRU-Net reflects the statistical property of GI and makes the residual of GI easy enough to learn. After training, our network obtains much better results than both the U-Net and DnCNN. The DRU-Net not only reconstructs high-quality images with an amount of details restored but also shows strong generalization ability on the test set.

The DRU-Net is made up of the up-CNN and down-CNN, which intends to learn the up-Res images and down-Res images, respectively. Here we define the up-Res and down-Res as follows

8 $$\begin{aligned} Res_{up}= & {} {\left\{ \begin{array}{ll} T(x,y) - T_{GI}(x,y), &{} T(x,y) > T_{GI}(x,y), \\ 0, &{} T(x,y) \le T_{GI}(x,y). \end{array}\right. } \end{aligned}$$

9 $$\begin{aligned} Res_{down}= & {} {\left\{ \begin{array}{ll} T_{GI}(x,y) - T(x,y), &{} T_{GI}(x,y) > T(x,y), \\ 0, &{} T_{GI}(x,y) \le T(x,y). \end{array}\right. } \end{aligned}$$

We replace the negative pixel values by zeros to make it more adaptive to convolution operation. We feed those highly corrupted GI images to networks as inputs and use the two residuals as targets separately. In this case, the training process can be written as

10 $$\begin{aligned} R_{up}= & {} \mathop {\arg \min }_{\theta \in \Theta _{up}} \sum _{i = 1}^{K} L(Res^i_{up}(x,y), R_{up_{\theta }} \{ T^i_{GI}(x,y) \}), \end{aligned}$$

11 $$\begin{aligned} R_{down}= & {} \mathop {\arg \min }_{\theta \in \Theta _{down}} \sum _{i = 1}^{K} L(Res^i_{down}(x,y), R_{down_{\theta }} \{ T^i_{GI}(x,y) \}), \end{aligned}$$

where $R_{up}$ denotes the up-CNN, and $R_{down}$ denotes the down-CNN. We also convert the floating-point number into an integer so that we can save the residuals as images without explicit loss of information. Now the reconstructed image can be expressed here as

12 $$\begin{aligned} O(x,y) = T_{GI}(x,y) + R_{up} \{ T_{GI}(x,y) \} - R_{down} \{ T_{GI}(x,y) \}. \end{aligned}$$

The MSRA10K dataset^38^ was used as the image of the object to train all three GIDLs in our study. We use the models of the U-Net and DnCNN in previous research^26,34,35^. We selected 5120 images and resized them into $128 \times 128$ pixels. After that, we divided them into batches and obtained corresponding GI images with the optimized simulation algorithm. According to Eqs. (8) and  (9), we calculated the up-Res and down-Res image paired up with corresponding GI images to train the DRU-Net. During the training period, Adam optimizer^39^ was adopted to minimize the mean square error (MSE). The batch size of the training set was 16. The learning rate was set to 0.00002, and we had the weight decay equal to 0.0003. We used PyTorch 1.3.1 and a laptop with an NVIDIA GTX 1080M graphic card to train our network.

## Results

**Figure 4:**
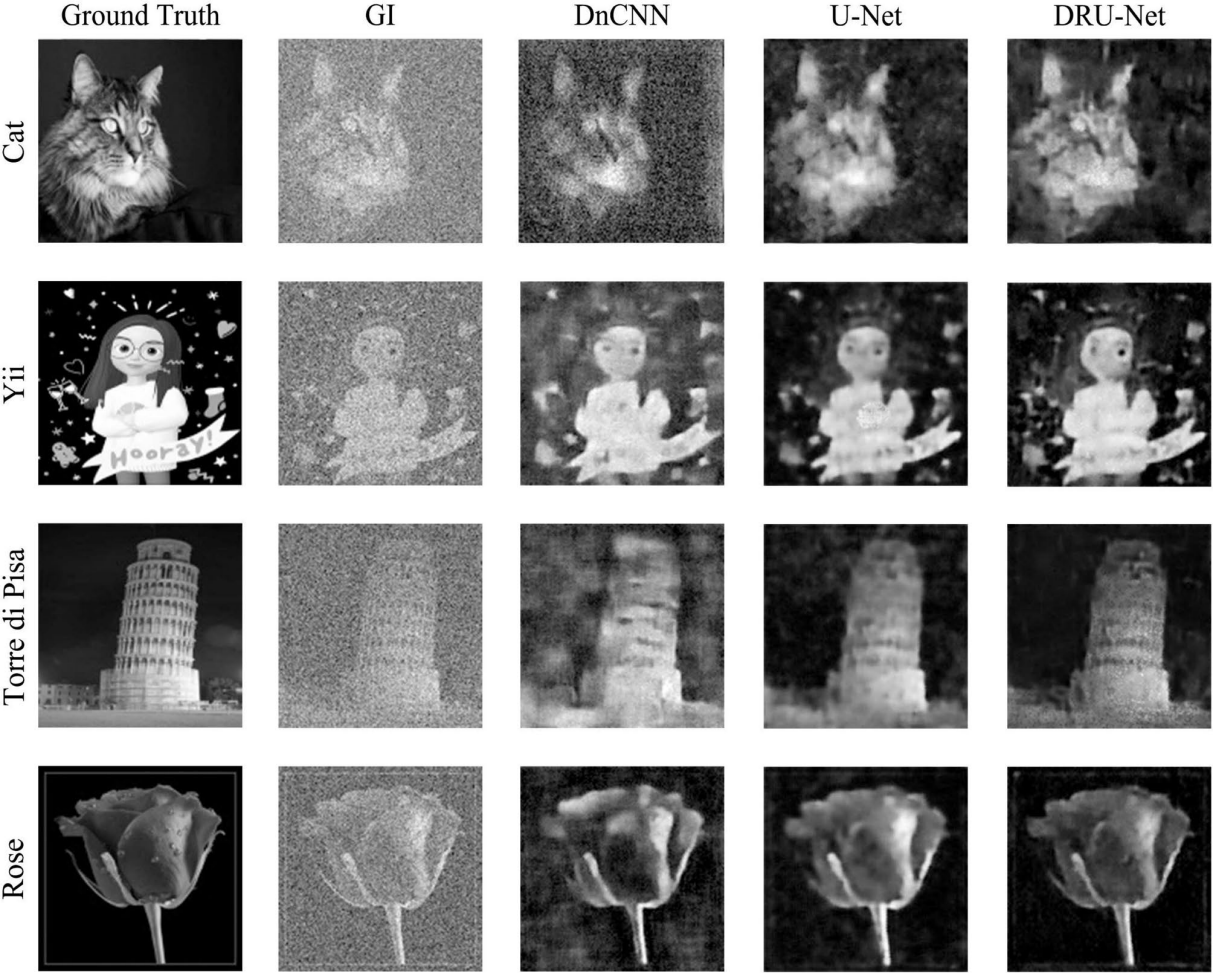
A comparison between the results of GI, DnCNN, U-Net and DRU-Net.

Figure 4 shows a comparison between the results of different GIDLs. The first column shows the four images of the object that are not included in the training set, while the second column shows the images reconstructed by GI simulation, the third shows the results produced by the DnCNN, the fourth shows the results of the U-Net and the fifth shows the results obtained by the DRU-Net.

As clearly shown in Fig. 4, both the U-Net and DRU-Net improve the quality of imaging significantly. The DnCNN performs not well, which is owing to the residual complexity of GI. It is obvious that our proposed DRU-Net achieves much better performance than other methods. In the image of Cat and Yii, more facial details are restored with the DRU-Net compared to the U-Net. In the image of Torre di Pisa, the gray value of DRU-Net is almost close to the image of the object, while the gray value of the U-Net is higher than the image of the object thus its dark background appears quite bright. As to the image of Rose, the image reconstructed using the DRU-Net is almost the same as the image of the object.

The DnCNN, U-Net and DRU-Net parameters for GI are shown in Table 1. The DRU-Net achieves the highest score under the measurement of both the PSNR and SSIM index and the lowest of the RMSE. The DnCNN performs poorly compared to the other two GIDLs. In the image of Yii, the RMSE of the DnCNN is higher than GI, which proves that the residual of GI is different from other image processing.

The SSIM index can evaluate image quality more accurately than the PSNR^26,40–42^. The average SSIM index of GI, DnCNN, U-Net, and DRU-Net are 0.183, 0.328, 0.482, and 0.555 respectively. The SSIM index of the image has been tripled with our proposed DRU-Net.

**Table 1:** PSNRs, SSIMs, and RMSEs for GI, DnCNN, U-Net, and DRU-Net. Higher PSNR and SSIM and lower RMSE present the higher quality image.

	Images	Cat	Yii	Torre di Pisa	Rose
PSNR	GI	9.463	9.549	12.011	10.228
	DnCNN	15.565	14.262	16.601	18.764
	U-Net	22.007	16.087	21.827	20.072
	DRU-Net	22.409	17.165	22.702	26.544
SSIM	GI	0.179	0.204	0.200	0.172
	DnCNN	0.239	0.338	0.312	0.340
	U-Net	0.535	0.411	0.564	0.482
	DRU-Net	0.584	0.460	0.606	0.684
RMSE	GI	10.564	10.453	10.508	10.746
	DnCNN	10.167	10.679	10.205	9.702
	U-Net	8.843	9.620	8.828	8.767
	DRU-Net	8.668	9.432	8.767	6.783

## Discussion

**Figure 5:**
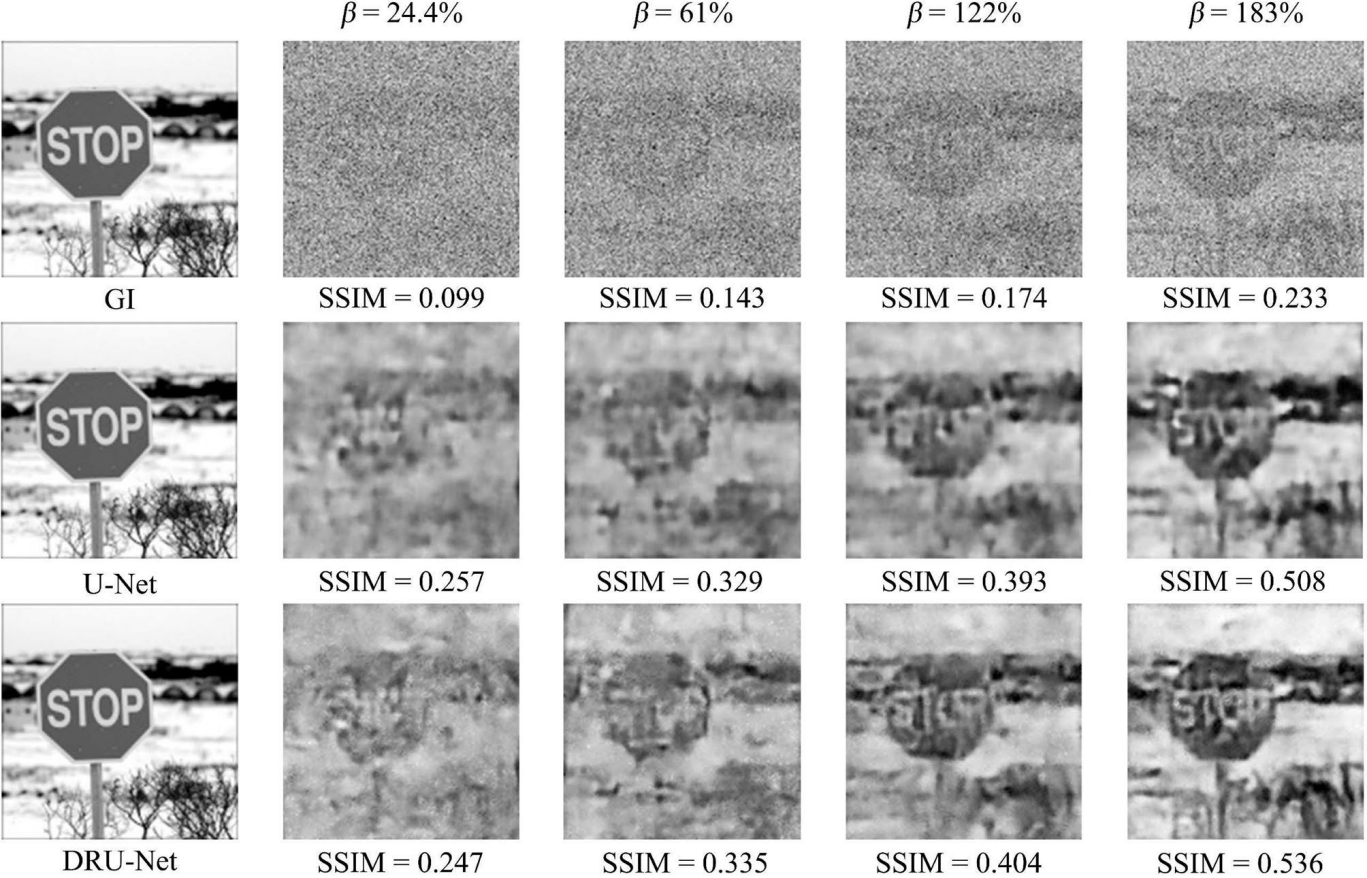
Network results under different sampling rate.

To further examine the robustness and generalization ability of the DRU-Net, we let them reconstruct images with different sampling rates, as $\beta = 24.4\%, 61\%, 122\%, 183\%$. The results are shown in Fig. 5. Here we mainly focus on the U-Net and DRU-Net, dismissing the results of DnCNN because it performs relatively weak compared with the other two networks. The first row shows the reconstructed images of GI; the second row shows that of the U-Net, and the third shows the results of the DRU-Net.

The quality of the reconstructed image continues to grow as the sampling rate increases. If $\beta$ equals to $24.4\%$, the reconstructed image of the road sign with the U-Net or DRU-Net are either highly blurred. When $\beta$ equals $61\%$, the profile of the object becomes clearer. However, the characters still cannot be reconstructed. When $\beta$ reaches as high as $183\%$, the word “STOP” can be recognized clearly. In the reconstructed images with the DRU-Net, more details of the water were restored than the image with U-Net. Nevertheless, the DRU-Net still cannot accomplish a very high-quality image since its PSNR is only 15.21dB. The noise in the process of GI has severely corrupted the image, which causes significant information loss. Since now, most GIDLs make use of supervised learning, and the GI process is taken as a denoising problem. In our future project, we plan to carry out the unsupervised learning methods, such as inpainting algorithm^43–45^ to “guess” the lost part of the object instead of learning since the significant information loss can hardly be viewed as denoising problem.

In summary, we propose the concept of batch in the pre-processing stage and optimize the simulation algorithm of GI. This allows more reliable simulation data with different sets of random patterns to be acquired within less time. The generalization ability of GIDL has been enhanced with the optimized simulation algorithm. However, we plan to involve the experimental research in our following study. Through careful analysis of the statistical property of GI, we further simplified the residual by dividing it into up-Res and down-Res images to form a new GIDL framework, namely the DR. Also, detailed comparisons of performances between the DnCNN, U-Net, and DRU-Net were discussed. DRU-Net showed much better reconstruction compared to the existing GIDLs. It has tripled the imaging quality of GI in the evaluation of the SSIM index.
